# Supplementary material for: Candidate Acetic Acid Bacteria Strains for Levan Production
Source: Polymers (Basel). 2022 May 13;14(10):2000. doi: 10.3390/polym14102000 (PMC9146431; doi:10.3390/polym14102000)
Supplement: Supplementary file 1 [file polymers-14-02000-s001.zip › polymers-1668357-supplementary.pdf]

Supplementary files

Table S1 Effect of sucrose concentration based on Pearson correlation index. R^2 values ≥ 0.6 were considered as positive correlation, while $R^2 \leq -0.6$ was considered as a negative correlation.

Species	Pearson index	P-value
N. chiangmaiensis NBRC 101099^T	0.65	0.0035
Ko. baliensis DSM 14400^T	0.63	0.0050
G. cerinus DSM 9533^T	-0.67	0.0023
G. frateurii DSM 7146^T	-0.55	0.0184
G. oxydans DSM 2343	-0.55	0.0172
K. xylinus DSM 2004	0.54	0.0214
K. xylinus DSM 6513^T	0.33	0.1867
K. xylinus K2G30=UMCC 2756	0.13	0.5949
K. hansenii DSM 5602^T	-0.46	0.0566
K. hansenii ATCC 53582	0.08	0.764
A. pasteurianus DSM 3509^T	0.34	0.1617
A. pasteurianus AB0220=UMCC 1754	0.48	0.0439

Table S2 Titratable acidity at low and high sucrose concentration at 48 and 96 h of incubation.

Species	70 g/L		250 g/L	
	48 h	96 h	48 h	96 h
N. chiangmaiensis NBRC 101099^T	0.63 ± 0.14	0.64 ± 0.01	0.86 ± 0.03	1.39 ± 0.06
Ko. baliensis DSM 14400^T	0.87 ± 0.06	1.13 ± 0.02	0.48 ± 0.03	2.58 ± 0.14
G. cerinus DSM 9533^T	0.78 ± 0.03	0.92 ± 0.04	0.07 ± 0.01	0.11 ± 0.01
G. frateurii DSM 7146^T	0.8 ± 0.08	0.91 ± 0.08	0.08 ± 0.01	0.09 ± 0.01
G. oxydans DSM 2343	0.41 ± 0.06	0.45 ± 0.06	0.08 ± 0	0.09 ± 0.01
K. xylinus DSM 2004	0.48 ± 0.03	0.39 ± 0.05	0.25 ± 0.03	0.31 ± 0.02
K. xylinus DSM 6513^T	0.35 ± 0.07	0.36 ± 0.07	0 ± 0	0.03 ± 0.01
K. xylinus K2G30=UMCC 2756	0.6 ± 0.17	0.61 ± 0.04	0.39 ± 0.03	0.21 ± 0.21
K. hansenii DSM 5602^T	0.32 ± 0.03	0.36 ± 0.03	0.08 ± 0.01	0.1 ± 0.01
K. hansenii ATCC 53582	0.3 ± 0.02	0.4 ± 0.06	0.18 ± 0.16	0.09 ± 0.01
A. pasteurianus DSM 3509^T	0.31 ± 0.06	0.27 ± 0.05	0.02 ± 0.02	0.05 ± 0.01
A. pasteurianus AB0220=UMCC 1754	0.45 ± 0.02	0.46 ± 0.23	0.16 ± 0.1	1.21 ± 0.57

*Values are mentioned as mean ± standard deviation.
